# Eye Motility Alterations in Retinitis Pigmentosa

**DOI:** 10.1155/2015/145468

**Published:** 2015-06-01

**Authors:** Raffaele Migliorini, Anna Maria Comberiati, Giovanni Galeoto, Manuela Fratipietro, Loredana Arrico

**Affiliations:** Department of Sense Organs, University of Rome “La Sapienza,” Via del Policlinico 155, 00161 Rome, Italy

## Abstract

*Purpose*. We evaluated a sample of individuals with retinitis pigmentosa (RP) with the aim of assessing the presence or absence of ocular motility (OM) disorders.* Materials and Methods*. We included 23 out of the 25 individuals from the sample (9 females and 14 males) with an average visual acuity of 6/10.* Results*. The cover test about the vertical deviation in near distance showed an r/l in 3.45% and an l/r in 6.9%. The assessment of OM showed that 39.1% of the sample had a severe hyperfunction of the IO of the right eye and a severe hyperfunction (34.5%) of the SO of the left eye; 21.8% had a moderate hypofunction of right SO with a moderate percentage of hypofunction of 17.5% for the SO of the left eye; 30.5%, however, showed a serious hypofunction of the SR of both eyes; 21.7% of the sample showed a hyperfunction in both eyes of the IR.* Conclusion*. This alteration, however, is not attributable to either a high refractive defect (medium-low myopia: −1 diopter ±3 SD) or to a severely impaired binocular vision (visual acuity, motor fusion, and stereopsis are normal or within a range of values commonly accepted). Therefore, the disorders of OM lead to a genetic origin.

## 1. Introduction

Retinitis pigmentosa (RP) is an inherited retinal disease characterized by the degeneration of photoreceptor rods and cones [1, 2]. In the majority of RP cases, there is a primary degeneration of rod photoreceptors with a secondary degeneration of cones. Thus, the typical RP is also described as a “rod-cone dystrophy” where photoreceptor rods are more affected than cones. This explains why patients show only a night blindness at first and a visual impairment later in daylight [3, 4].

In some cases, the clinical presentation is “cone-rod dystrophy” type with a predominant involvement of the cones or the central retinal photoreceptors. The decrease of visual acuity consequently predominates over visual field loss [1, 5].

The worldwide prevalence of retinitis pigmentosa is about 1 in 4,000 healthy people for a total of over 1 million people affected. In the USA it is about 1 : 3500–1 : 4000 with significant differences between the various states; in Switzerland 1 : 7000; China 1 : 4016; Norway 1 : 4440; in Israel 1 : 4500 [6].

The disease can be inherited as autosomal-dominant (about 30–40% of cases), autosomal-recessive (50–60%), or X-linked (5 to 15%) [6]. On the basis of this data, an observational study was performed on a sample of individuals suffering from retinitis pigmentosa. In the literature, we did not find any other studies concerning the alterations of ocular motility in retinitis pigmentosa.

The purpose of the study was to highlight the presence or absence of eye movement disorders in a genetically determined disease like RP while excluding all those influential factors used in recruiting binocular vision and the development of abnormal ocular motility such as high refractive errors [7], visual acuity < 6/10, pituitary adenomas, and related eye diseases in order to show the type of muscle alteration, if present, and then reconnect it to a genetic cause like RP.

## 2. Materials and Methods 

Our sample consisted of 50 eyes (25 individuals) with retinitis pigmentosa from the Center for Pediatric Ophthalmology at the Eye Clinic of the Policlinico Umberto I, University of Rome La Sapienza. Patients were evaluated by the same examiner, and after a careful history and a thorough eye examination of both the anterior segment and posterior segment with indirect ophthalmoscopy (Schepens), they were put through the following orthotic tests:

- visual acuity or visual acuity;
- corneal reflex (CR);
- stereopsis test;
- cover test (CT);
- ocular motility (OM);
- convergence objective.

The inclusion criteria of our study were as follows.

- Age between 6 and 80.
- Patients with the typical form of nonsyndromic RP and patients with syndromes that are associated with various types of pigmentary retinopathy (e.g., Usher syndrome, Cockayne syndrome, Best's disease, etc.).
- Patients with suspected RP: by careful history the presence of RP was discovered in other family members (i.e., brother, cousin, and grandparent).
- Visual acuity between 6/10 and a maximum of 10/10 (via the Snellen optotype).

The exclusion criteria were as follows.

- Preschool age (from 1 to 5 years).
- Patients who had undergone ocular surgery.
- Patients with systemic, vascular, and neurodegenerative disease (e.g., the “multiple sclerosis”) that can affect the orthoptic assessment.
- Patients with visual field (CV) electronic Humphrey < 10°.
- Visual acuity below 6/10.
- Presence of pituitary adenomas.

In the evaluation of ocular motility, we considered the 12 extraocular muscles of both eyes in different positions of gaze. We have assigned a gradient equal to 0 in the case of normal ocular motility, +1 in the case of mild hyperfunction, +2 in the presence of moderate, and +3 in the case of severe hyperfunction. The same score was given in the case of hypofunction of the contralateral synergist muscle but with a negative value. A statistical analysis of this pilot study was performed by a Pearson correlation between the contralateral synergist muscles: ri SR-le SO, ri IR-le GO, le SR-riSO, and le IR-ri GO.

## 3. Results

The sample consisted of 25 individuals but was reduced to 23 individuals, because two patients reporting a severe mental retardation (IQ < 40) were unable to cooperate with most important orthotic tests such as an accurate assessment of ocular motility.

**Figure 1:**
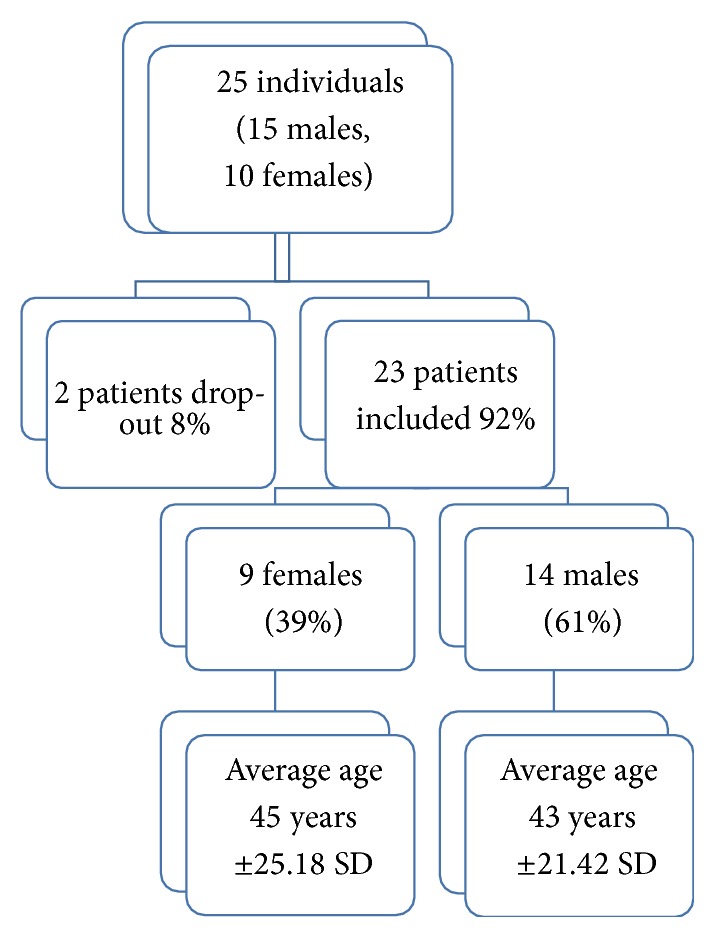
Stratification of the sample by age and sex.

Thus, out of the 25 individuals, 23 have been included with 9 females and 14 males. The average age for women was 45 years ±25.18 SD while the average age found in men was 43 age ±21.42 SD (Figure 1).

Of the 23 patients included, 69.5% were suffering from typical retinitis pigmentosa (rod cone dystrophy) and 4.3% by RP atypical (cone-rod dystrophy). Patients with syndromes associated with RP (such as S. Cockayne and S. Uscher) showed the same percentage of disease incidence of 4.3%. Among macular dystrophy, Best's disease was found in 13.3%: 4 patients belonging to the same family (father with sons and grandson). We also found a case report with Cones dystrophy (incidence 4.3%).

**Figure 2:**
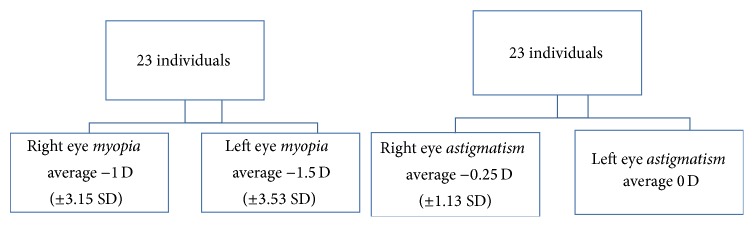
Evaluation of refractive errors. The data showed refractive errors (mild myopia and low astigmatism) not able to alter binocular vision and therefore the ocular motility of the examined patients.

Among refractive defects found in our sample, we found the presence of myopia with an average of 1 diopter ±3.15 SD for the right eye and 1.5 diopter ±3.53 SD for the left eye. Astigmatism, compared to myopia, was not statistically significant in both eyes. In fact, there has only been an average of −0.25 for the right eye with SD equal to 1.13. This data showed refractive errors (myopia of middle-grade and low, irrelevant, astigmatism) that are not able to affect binocular vision and therefore the ocular motility of the tested patients (Figure 2).

In the evaluation of visual acuity in tenths by the Snellen optotype, it is appropriate to mention the presence of an average of 6/10 ± SD of 2.9 for the right eye and 3.2 for the left eye. This data does not show a significant reduction in visual acuity that can hinder the development of binocular vision. The latter has also been evaluated through some stereoscopic tests (Lang I and II) that show full results in 22 of the 23 patients. In only one patient (a child with exotropia) and when detecting only the star image, the three-dimensional sense was absent. We can say that the development of binocular vision is not compromised due to alack in the whole sample of diplopia guarantor of an optimal sensorial fusion. Thereafter in the study of fusional amplitude, except for the young exotropic girl, the rest of the sample showed normal motor fusion values.

In the evaluation of ocular motility, we took into account the different positions of gaze: primary position, left, right, up, up right and left, down, down left, and right. It was found that no patient had an alteration of the medial rectus muscle, and the lateral rectus was present in the right and left side sight. It is necessary, however, to highlight the presence of the alteration in 50% of the sample in various degrees of ocular motility regarding the small oblique muscles, large oblique, rectus superior, and inferior rectus. In the upper left gaze, there has been a severe grade (score = +3) hyperfunction of the small oblique muscle (SO) of the right eye in 39.1% of cases compared with a normal ocular motility (score = 0) in 34.7% of our sample. We also verified a moderate hyperfunction (score = +2) in 17.4% of cases and an equal incidence of 4.4% of the cases in both the mild hyperfunction (score = +1) and the severe hypofunction (score = −3) of the muscle. Besides finding a severe hyperfunction of the right SO in the look upward and left, we found in 30.5% of the cases studied the presence of a severe hypofunction of the contralateral synergist, namely, the superior rectus of the left eye. In addition, out of the 23 individuals, we also found a normal motility in 34.8% of cases, a moderate hypofunction in 21.8% of cases, a severe hyperfunction in 8.6%, and a mild hypofunction in 4.3% for the superior rectus muscle of the left eye. In the ocular motility evaluation, we also took into consideration the position of gaze at the bottom left. In that position, we found the presence of a hypofunction of the great oblique muscle of the right eye of a moderate degree (score = −2). In 21.8% of the 23 individuals considered and, on the other hand, in 61% of cases, the ocular motility was normal. With the same percentage of the total 4.3%, the large oblique muscle of the right eye showed a moderate hyperfunction, a mild hyperfunction, and a severe hypofunction. In versions, considering the gaze of eyes to the bottom left, the grand oblique muscle of the right eye enters to play together with the contralateral synergist, that is, the inferior rectus muscle of the left eye. Next to a moderate hypofunction of the right GO, we noted the presence of an alteration of the OM of different degrees in left IR. In fact, the lower rectus left eye showed, in 21.7% of cases, a severe hyperfunction this time (score = +3). In addition, our sample showed an optimum operation of the inferior rectus muscle of the left eye in 60.9% and a moderate hypofunction in 8.7% with the same percentage of 4.4% of cases, and the IR of the left eye presented a mild and moderate hyperfunction.

Another gaze direction where we found an alteration of ocular motility is the one in the top right. We noticed the presence of a severe hypofunction of the right superior rectus muscle (score = −3) observed in 30.5% of cases compared to 39.1% of cases who reported a normal function of this muscle. Furthermore, in 17.5% of cases, the SR of the right eye showed a Moderate hypofunction, 8.6% a severe hyperfunction, and a mild hypofunction of only 4.3%.

For the diagnostic position of gaze in the top right, as well as, having experienced a severe hypofunction of the SR of the right eye, we also detected a hyperfunction of the same grade (score = +3) of the contralateral synergist muscle, that is, the small oblique of left eye with an incidence in 34.8% of cases. In 39.1% of the 23 individuals, however, the operation of the SO of the left eye was optimal while 13% had a moderate hyperfunction, 8.7% a severe hypofunction, and 4.4% a mild hyperfunction.

Finally, we found the presence of an alteration of ocular motility in the gaze to the lower right. This alteration was found in the inferior rectus muscle of the right eye that showed a severe hyperfunction in 21.7% of cases and the same percentage of incidence equal to 4.4% of the cases for a mild hyperfunction and moderate hypofunction. Instead in 69.5% of cases, the inferior rectus muscle of the right eye showed a normal operation. In the diagnostic position in the lower right corner, as well as in the gaze to the lower left, we noted the presence of an alteration in ocular motility of different degrees for contralateral synergist muscles. In fact, a severe hyperfunction (score = +3) of the IR of the right eye is associated with a moderate hypofunction (score = −2) of large oblique muscle of the left eye in 17.5% of cases. Taking into consideration the position of gaze, the GO muscle of the left eye worked normally in 69.6% of cases, while it showed a severe hypofunction and moderate hyperfunction with the same percentage of 4.3% (Table 1). Through a Pearson correlation, we can say that the contralateral synergist muscles being compared from the evaluation of ocular motility are inversely correlated, and this allows us to state that there is a direct relationship between the retinitis pigmentosa and ocular motility disorders (Table 2).

Prime Position Muscle Hypofunction Mild Hypofunction Moderate Hypofunction Severe Normal Hyperfunction Mild Hyperfunction Moderate Hyperfunction Severe.

In the upper left: right Inferior Oblique 0 0 4.4% 34.7% 4.4% 17.4% 39.1%—left Superior Rectus 4.3% 21.8% 30.5% 34.8% 0 0 8.6%.

In the upper right: right Superior Rectus 4.3% 17.5% 30.5% 39.1% 0 0 8.6%—left Inferior Oblique 0 8.7% 0 39.1% 4.4% 13% 34.8%.

In the bottom left: right Superior Oblique 4.3% 21.8% 4.3% 61% 0 4.3% 4.3%—left Inferior Rectus 0 8.7% 60.9% 0 4.4% 4.4% 21.7%.

In the bottom right: left Superior Oblique 4.3% 17.5% 4.3% 69.6% 0 4.33% 0—right Inferior Rectus 0 4.4% 0 69.5% 4.4% 0 21.7%.

**Figure 3:**
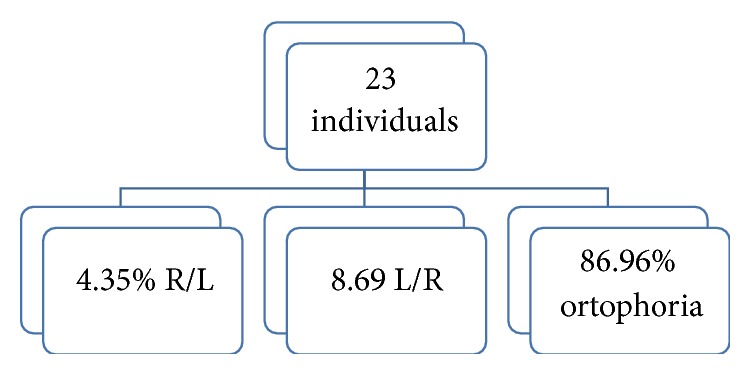
Cover test: evaluation of vertical deviation for near (with or without corrective lenses).

Using the test cover, the evaluation of 25 patients examined with and without corrective lenses for near and evaluated for near (by observing a target light) and for far (indicating a letter of the optotype in relation to the patient's visual acuity) showed interesting data. Indeed, in our sample we have found the presence of a vertical deviation in 4 patients; among these, 2 (a patient with RP and one with S. of Uscher) reported a deviation L/R with and without the lens, and the other 2 (a typical patient with RP and the other with atypical RP) that reported a deviation R/L. Considering the vertical deviation of the cover test examination, we found a L/R with an incidence of 8.69% and a R/L in 4.35% of cases tested for near with and without corrective lenses (Figure 3). In the evaluation, however, for far (with and without lens), the cover test has highlighted the presence of a R/L in 4.35% of the 23 individuals observed; while in almost all of our sample (95.65% of cases) ortoforia was present. This vertical deviation could justify the alteration of ocular motility observed in our RP sample, but the percentage is statistically insignificant.

**Figure 4:**
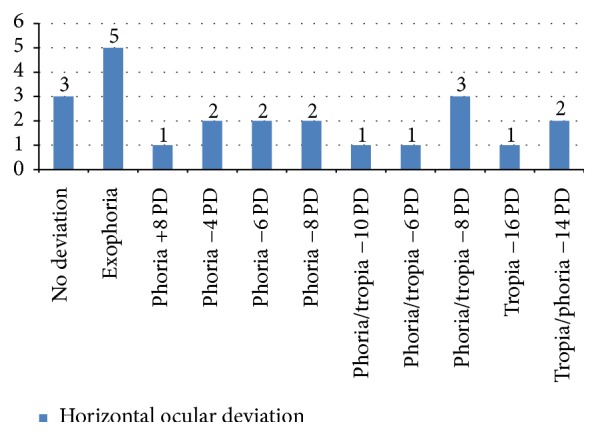
Cover test: evaluation of horizontal deviation for near with the lens.

**Figure 5:**
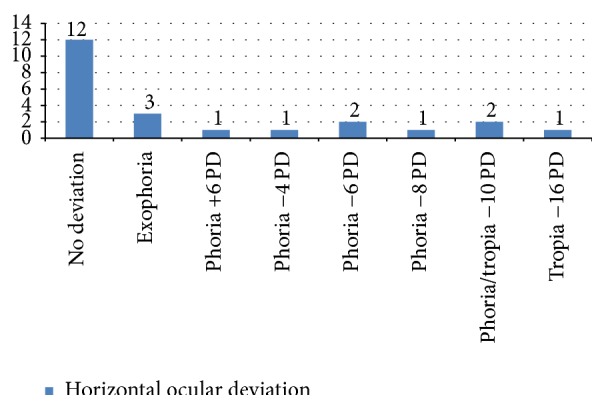
Cover test: evaluation of the horizontal deviation for near without lens.

The data concerning the horizontal deviation highlighted with alternate cover test in the 25 patients evaluated with and without corrective lenses for near and far were 3 individuals during the dissociation cover test for near with lens showed no changes in refixation (orthoforic subjects), while 5 patients had a recovery movement of the eye just discovered from the outside inwards (exoforia). In cases of deviation greater than 2 PD, the angle of deviation has been measured with a prism with a base opposite to the direction of the deviation and assigning the + sign in the case of ESO and the − sign in the case of EXO. The cover-uncover was essential to establish the quality of the deviation: if it is phoria or tropiaor if there is a recovery of deviation (phoria-tropia) and what is the fixing eye. We noted only one case of exotropia of the left eye of 16 PD and two cases of tropia-phoria of −14 PD. Only in these 3 cases has there been an alteration of ocular motility directly proportional to the amount of deviation in the primary position. Moreover, the presence of a case of esoforia of 8 PD and two cases of phoria-tropia, one of −10 PD and the other one of −6 PD, were found. In 6 patients, there was exoforia ranging from 4 PD to 8 PD. Finally, 3 patients showed a phoria-tropia of −8 PD. (Figure 4). In the same patients, CT was performed always for near but without corrective lenses. We found that 12 patients had no movements of refixation, 3 patients were exoforical, and 2 patients showed a phoria-tropia of −10 PD. There was only one case of a 15-year-old girl who, even without corrective lenses, had a tropia (−16 PD). Additionally, 3 patients had, respectively, anesoforia of 6 PD and anexoforia of 4 PD and 8 PD (Figure 5). Finally, in the CT evaluation with or without a lens for far, almost all of our sample did not show any significant horizontal deviation. In fact, the CT for distance with lens showed that 22 individuals did not show any deviation, and a tropia-phoria of −12 PD was found in a 15-year-old deaf and dumb girl who presented an exotropia to corneal reflexes. While always considering the CT for distance but without corrective lenses, we found in the same 15-year-old child a tropia-phoria of −14 PD and 3 cases of exoforia. Most of our sample, however, showed no deviation (19 individuals out of 23).

## 4. Conclusions 

The sample recruited showed a significant proportion of patients with pure retinitis pigmentosa (69.5%), and the remaining part was suffering from associated syndromes. From the assessment of ocular motility, it was evident that in no patient had an alteration of the medial rectus and lateral rectus muscles in left and right side gaze.

It is instead essential to mention the presence of an ocular motility alteration for the small oblique, large oblique, superior rectus, and inferior rectus muscles in 50% of the sample.

39.1% of the sample has a severe hyperfunction of the small oblique muscle of the right eye and a severe hyperfunction (34.5%) of the small oblique of the left eye; 21.8% have a moderate hypofunction of the large oblique muscle of the right eye with a moderate percentage of hypofunction of 17.5% for the great oblique of the left eye; 30.5% of the sample has, however, a percentage of 30.5% of severe hypofunction of both eyes' superior rectus muscles; 21.7% of the sample showed a hyperfunction of the inferior rectus muscle in both eyes.

The results show that there is an impaired motility in 50% of patients in this inherited disorder. This alteration of the ocular motility is not, however, due either to a high refractive defect (size medium-low myopia: −1 diopter ±3 SD) or to a binocular vision severely impaired (visual acuity, motor fusion, and stereopsis are normal or within commonly accepted limit values). These ocular motility disorders are ascribed to a genetic origin factor. In fact, since RP is a genetically determined disease, the absence of eye movement disorders in the other 50% of the sample could be linked to the different penetrance of the disease that determines the existence of healthy carriers. Therefore, the results of this study indicate that, in patients with RP, there is an alteration of ocular motility and this indicates that a careful orthotic screening may allow a further contribution to an early diagnosis especially in those cases of RP with family history and in healthy carriers.

## Figures and Tables

**Table 1:** Evaluation of ocular motility.

Primary position	Muscle	Ipofunction slight	Ipofunction moderate	Ipofunction severe	Normal motility	Iperfunction slight	Iperfunction moderate	Iperfunction serious
In the left upward	Inferior oblique of the right eye	0	0	4.4%	34.7%	4.4%	17.4%	39.1%
	Superior rectus of the left eye	4.3%	21.8%	30.5%	34.8%	0	0	8.6%

In the right upward	Superior rectus of the right eye	4.3%	17.5%	30.5%	39.1%	0	0	8.6%
	Inferior oblique of the left eye	0	8.7%	0	39.1%	4.4%	13%	34.8%

In the left downward	Superior oblique of the right eye	4.3%	21.8%	4.3%	61%	0	4.3%	4.3%
	Inferior rectus of the left eye	0	8.7%	0	60.9%	4.4%	4.4%	21.7%

In the right downward	Superior oblique of the left eye	4.3%	17.5%	4.3%	69.6%	0	4.33%	0
	Inferior rectus of the right eye	0	4.4%	0	69.5%	4.4%	0	21.7%

**Table 2:** Pearson correlation in ocular motility.

Right SR—Left IO	−0.99
Right IR—Left SO	−0.97
Left SR—Right IO	−0.74
Left IR—Right SO	−0.96
